# Interval-valued fuzzy $\phi$-tolerance competition graphs

**DOI:** 10.1186/s40064-016-3463-z

**Published:** 2016-11-15

**Authors:** Tarasankar Pramanik, Sovan Samanta, Madhumangal Pal, Sukumar Mondal, Biswajit Sarkar

**Affiliations:** 1 Department of Mathematics, Khanpur Gangche High School (H.S.), Khanpur, Pandua, India; 2 Department of Industrial and Management Engineering, Hanyang University, Ansan, Gyeonggi 15588 South Korea; 3 Department of Applied Mathematics with Oceanology and Computer Programming, Vidyasagar University, Midnapore, 721102 India; 4 Department of Mathematics, Raja N.L. Khan Women’s College, Midnapore, 721102 India

**Keywords:** Competition, Tolerance, Interval-valued fuzzy graphs

## Abstract

This paper develops an interval-valued fuzzy $\phi$-tolerance competition graphs which is the extension of basic fuzzy graphs and $\phi$ is any real valued function. Interval-valued fuzzy $\phi$-tolerance competition graph is constructed by taking all the fuzzy sets of a fuzzy $\phi$-tolerance competition graph as interval-valued fuzzy sets. Product of two IVFPTCGs and relations between them are defined. Here, some hereditary properties of products of interval-valued fuzzy $\phi$-tolerance competition graphs are represented. Application of interval-valued fuzzy competition graph in image matching is given to illustrate the model.

## Background

Graphs can be considered as the bonding of objects. To emphasis on a real problem, those objects are being bonded by some relations such as, friendship is the bonding of pupil. If the vagueness in bonding arises, then the corresponding graph can be modelled as fuzzy graph model. There are many research available in literature like Bhutani and Battou (2003) and Bhutani and Rosenfeld (2003).

Competition graph was defined in Cohen (1968). In ecology, there is a problem of food web which is modelled by a digraph $\overrightarrow{D}=(V,\overrightarrow{E})$. In food web there is a competition between species (members of food web). A vertex $x\in V(\overrightarrow{D})$ represents a species in the food web and arc $\overrightarrow{(x,s)}\in \overrightarrow{E}(\overrightarrow{D})$ means that *x* kills the species *s*. If two species *x* and *y* have common prey *s*, they will compete for *s*. Based on this analogy, Cohen (1968) defined a graph model (competition graph of a digraph), which represents the relationship of competition through the species in the food web. The corresponding undirected graph $G=(V,E)$ of a certain digraph $\overrightarrow{D}=(V, \overrightarrow{E})$ is said to be a competition graph $C(\overrightarrow{D})$ with the vertex set *V* and the edge set *E*, where $(x,y)\in E$ if and only if there exists a vertex $s\in V$ such that $\overrightarrow{(x,s)},\overrightarrow{(y,s)}\in \overrightarrow{E(\overrightarrow{D})}$ for any $x,y\in V,\, (x\ne y)$.

There are several variations of competition graphs in Cohen’s contribution (Cohen 1968). After Cohen, some derivations of competition graphs have been found in Cho et al. (2000). In that paper, *m*-step competition graph of a digraph was defined. The *p*-competition graph of a digraph is defined in Kim et al. (1995). The p-competition means if two species have at least *p*-common preys, then they compete to each other.

In graph theory, an intersection graph is a graph which represents the intersection of sets. An interval graph is the intersection of multiset of intervals on real line. Interval graphs are useful in resource allocation problem in operations research. Besides, interval graphs are used extensively in mathematical modeling, archaeology, developmental psychology, ecological modeling, mathematical sociology and organization theory.

Tolerance graphs were originated in Golumbic and Monma (1982) to extend some of the applications associated with interval graphs. Their original purpose was to solve scheduling problems for arrangements of rooms, vehicles, etc. Tolerance graphs are generalization of interval graphs in which each vertex can be represented by an interval and a tolerance such that an edge occurs if and only if the overlap of corresponding intervals is at least as large as the tolerance associated with one of the vertices. Hence a graph $G = (V,E)$ is a tolerance graph if there is a set $I = \{I_v{:}\,v \in V\}$ of closed real intervals and a set $\{T_v{:}\,v \in V\}$ of positive real numbers such that $(x,y) \in E$ if $|I_x\cap I_y| \ge {{\rm min}} \{ T_x,T_y\}$. The collection <$I,T$> of intervals and tolerances is called tolerance representation of the graph *G*.

Tolerance graphs were used in order to generalize some well known applications of interval graphs. In Brigham et al. (1995), tolerance competition graphs was introduced. Some uncertainty is included in that paper by assuming tolerances of competitions. A recent work on fuzzy *k*-competition graphs is available in Samanta and Pal (2013). In the paper, fuzziness is applied in the representation of competitions. Recently Pramanik et al. defined and studied fuzzy $\phi$-tolerance competition graph in Pramanik et al. (2016). But, fuzzy *phi*-tolerance targets only numbers between 0 and 1, but interval-valued numbers are more appropriate for uncertainty. Other many related works are found in Pramanik et al. (2014) and Samanta and Pal (2015).

**Fig. 1:**
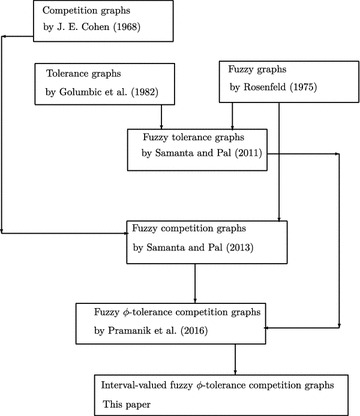
Flow-chart of the research

After (Rosenfeld 1975), the fuzzy graph theory increases with its various types of branches. Using these concept of fuzzy graphs, Koczy (1992) discussed fuzzy graphs to evaluate and to optimize any networks. Samanta and Pal (2013) showed that fuzzy graphs can be used in competition in ecosystems. After that, they introduced some different types of fuzzy graphs (Samanta and Pal 2015; Samanta et al. 2014). Bhutani and Battou (2003) and Bhutani and Rosenfeld (2003) discussed different arcs in fuzzy graphs. For further details of fuzzy graphs, readers may look in Mathew (2009), Mordeson and Nair (2000) and Pramanik et al. (2014). Applications of fuzzy graph include data mining, image segmentation, clustering, image capturing, networking, communication, planning, scheduling, etc. In this paper, interval valued fuzzy $\phi$-tolerance competition graph is introduced. Some relations on product of interval valued $\phi$-tolerance competition graphs are established. The authors’ contributions to develop competition graphs and tolerance graphs are listed in the Table 1. Also, the flow chart of the research contribution towards this research is given in Fig. 1.

**Table 1:** Contributions of the authors towards interval valued $\phi$-tolerance competition graphs

Authors	Year	Contributions
Cohen (1968)	1968	Introduced competition graphs
Kauffman (1973)	1973	Defined fuzzy graphs
Rosenfeld (1975)	1975	Modified the concept of fuzzy graphs given by Kauffman (1973)
Golumbic and Monma (1982)	1982	Established the concept of tolerance graphs
Cho et al. (2000)	2000	Defined *m*-step competition graphs
Samanta and Pal (2011)	2011	Introduced fuzzy tolerance graphs
Samanta and Pal (2013)	2013	Proposed the concept of fuzzy competition graphs
Pramanik et al. (2016)	2016	Advanced the idea of fuzzy $\phi$-tolerance competition graphs and defined $\phi$-tolerance competition graphs
This paper	–	Introduction of interval valued fuzzy $\phi$-tolerance competition graphs

## Preliminaries

A function $\alpha {:}\,X\rightarrow [0,1]$, called the *membership function* defined on the crisp set *X* is said to be a *fuzzy set* $\alpha$ on *X*. The *support* of $\alpha$ is ${{\mathrm{supp}}}(\alpha ) =\{x\in X| \alpha (x)\ne 0\}$ and the *core* of $\alpha$ is ${\mathrm{core}}(\alpha ) = \{x\in X| \alpha (x)=1\}$. The *support length* is $s(\alpha )=|{{\mathrm{supp}}}(\alpha )|$ and the *core length* is $c(\alpha )=|{{\mathrm{core}}}(\alpha )|$. The height of $\alpha$ is $h(\alpha ) =\max \{\alpha (x)| x\in X\}$. The fuzzy set $\alpha$ is said to be *normal* if $h(\alpha )=1$.

A *fuzzy graph* with a non-void finite set *V* is a pair $G = (V, \sigma ,\mu )$, where $\sigma {:}\,V \rightarrow [0,1]$ is a fuzzy subset of *V* and $\mu {:}\,V\times V\rightarrow [0,1]$ is a fuzzy relation (symmetric) on the fuzzy subset $\sigma$, such that $\mu (x,y) \le \sigma (x) \wedge \sigma (y)$, for all $x,y\in V$, where $\wedge$ stands for minimum. The *degree* of a vertex *v* of a fuzzy graph $G = (V, \sigma ,\mu )$ is $\displaystyle d(v)=\sum \nolimits _{u\in V-\{v\}}\mu (v,u)$. The *order* of a fuzzy graph *G* is $\displaystyle O(G)=\sum \nolimits _{u\in V}\sigma (u)$. The *size* of a fuzzy graph *G* is $\displaystyle S(G)=\sum \mu (u,v)$.

Let ${\mathcal {F}}=\{\alpha _1,\alpha _2,\ldots , \alpha _n\}$ be a finite family of fuzzy subsets on a set *X*. The fuzzy intersection of two fuzzy subsets $\alpha _1$ and $\alpha _2$ is a fuzzy set and defined by $\alpha _1\wedge \alpha _2=\left\{ \min \{\alpha _1(x),\alpha _2(x)\}|x\in X\right\}$. The union of two fuzzy subsets $\alpha _1$ and $\alpha _2$ is a fuzzy set and is defined by $\alpha _1\vee \alpha _2=\left\{ \max \{\alpha _1(x),\alpha _2(x)\}|x\in X\right\}$. $\alpha _1\le \alpha _2$ for two fuzzy subsets $\alpha _1$ and $\alpha _2$, if $\alpha _1(x)\le \alpha _2(x)$ for each $x\in X$.

The fuzzy intersection graph of ${\mathcal {F}}$ is the fuzzy graph $Int({\mathcal {F}})=(V, \sigma ,\mu )$, where $\sigma {:}\,{\mathcal {F}}\rightarrow [0,1]$ is defined by $\sigma (\alpha _i)=h(\alpha _i)$ and $\mu {:}\,{\mathcal {F}}\times {\mathcal {F}} \rightarrow [0,1]$ is defined by

$$\begin{aligned} \mu (\alpha _i,\alpha _j)=\left\{ \begin{array}{ll} h(\alpha _i\wedge \alpha _j), &{}\quad {\text{if}}\, i\ne j\\ 0, &{}\quad {\text{if}}\, i=j. \end{array}\right. \end{aligned}$$

Here, $\mu (\alpha _i,\alpha _i)=0$ for all $\alpha _i$ implies that the said fuzzy graph is a loop less fuzzy intersection graph and the fuzzy graph has no parallel edges as $\mu$ is uniquely defined.

Let us consider a family of fuzzy intervals ${\mathcal {F}}_{\mathcal {I}}=\{{\mathcal {I}}_1, {\mathcal {I}}_2, \ldots , {\mathcal {I}}_n\}$ on *X*. Then the fuzzy interval graph is the fuzzy intersection graph of these fuzzy intervals ${\mathcal {I}}_1, {\mathcal {I}}_2, \ldots , {\mathcal {I}}_n$.

Fuzzy tolerance of a fuzzy interval is denoted by ${\mathcal {T}}$ and is defined by an arbitrary fuzzy interval, whose core length is a positive real number. If the real number is taken as *L* and $|i_k-i_{k-1}|=L$, where $i_k,i_{k-1}\in R$, a set of real numbers, then the fuzzy tolerance is a fuzzy set of the interval $[i_{k-1},i_k]$.

The fuzzy tolerance graph ${\mathcal {G}}=(V,\sigma ,\mu )$ as the fuzzy intersection graph of finite family of fuzzy intervals ${\mathcal {I}}=\{{\mathcal {I}}_1,{\mathcal {I}}_2,\ldots , {\mathcal {I}}_n\}$ on the real line along with tolerances ${\mathcal {T}}=\{{\mathcal {T}}_1,{\mathcal {T}}_2,\ldots ,{\mathcal {T}}_n\}$ associated to each vertex of $v_i\in V$, where, $\sigma {:}\, V\rightarrow [0,1]$ is defined by $\sigma (v_i)=h({\mathcal {I}}_i)=1$ for all $v_i\in V$ and $\mu {:}\, V\times V\rightarrow [0,1]$ is defined by

$$\begin{aligned} \mu (v_i,v_j)=\left\{ \begin{array}{ll} 1, &{}\quad {\text { if }}\, c({\mathcal {I}}_i\cap {\mathcal {I}}_j)\ge \min \{c({\mathcal {T}}_i),c({\mathcal {T}}_j)\}\\ \frac{s({\mathcal {I}}_i\cap {\mathcal {I}}_j)-\min \{s({\mathcal {T}}_i), s({\mathcal {T}}_j)\}}{s({\mathcal {I}}_i\cap {\mathcal {I}}_j)}h({\mathcal {I}}_i\cap {\mathcal {I}}_j), &{}\quad {\text { else if }}\,s({\mathcal {I}}_i\cap {\mathcal {I}}_j)\ge \\ &{}\quad \min \{s({\mathcal {T}}_i),s({\mathcal {T}}_j)\}\\ 0, &{}\quad {\text { otherwise}}. \end{array}\right. \end{aligned}$$

Fuzzy interval digraph is a directed fuzzy interval graph, whose edge membership function need not to be symmetric.

An *interval number* (Akram and Dudek 2011) *D* is an interval $[a^-, a^+]$ with $0\le a^-\le a^+\le 1$. For two interval numbers $D_1=[a_1^-,a_1^+]$ and $D_2=[a_2^-,a_2^+]$, the following properties are defined:

1. $$D_1+D_2=[a_1^-,a_1^+]+[a_2^-,a_2^+]=[a_1^-+a_2^- -a_1^-\cdot a_2^-, a_1^+ +a_2^+ - a_1^+\cdot a_2^+],$$
2. $$\min \{D_1,D_2\}=[\min \{a_1^-,a_2^-\}, \min \{a_1^+,a_2^+\}],$$
3. $$\max \{D_1,D_2\}=[\max \{a_1^-,a_2^-\}, \max \{a_1^+,a_2^+\}],$$
4. $$D_1\le D_2 \Leftrightarrow a_1^-\le a_2^-$$ and $$a_1^+\le a_2^+$$,
5. $$D_1=D_2 \Leftrightarrow a_1^-= a_2^-$$ and $$a_1^+= a_2^+$$,
6. $$D_1<D_2 \Leftrightarrow D_1\le D_2$$ and $$D_1\ne D_2$$,
7. $$kD_1=[ka_1^-, ka_2^+]$$, where $$0\le k\le 1$$.

An interval-valued fuzzy set *A* on a set *X* is a function $\mu _A{:}\, X\rightarrow [0,1]\times [0,1]$, called the membership function, i.e. $\displaystyle \mu _A(x)=[\mu _A^-(x), \mu _A^+(x)]$. The support of *A* is ${{\mathrm{supp}}}(A)=\{x\in X|\mu _A^-(x)\ne 0\}$ and the core of *A* is ${{\mathrm{core}}}(A)=\{x\in X | \mu _A^-(x)=1\}$. The support length is $s(A)=|{{\mathrm{supp}}}(A)|$ and the core length is $c(A)=|{{\mathrm{core}}}(A)|$. The height of *A* is $\displaystyle h(A)=\max \{\mu _A (x)|x\in X\}=[\max \{\mu _A^-(x)\}, \max \{\mu _A^+(x)\}], \forall x\in X$. Let $F=\{A_1, A_2, \ldots , A_n\}$ be a finite family of interval-valued fuzzy subsets on a set *X*. The fuzzy intersection of two interval-valued fuzzy sets (IVFSs) $A_1$ and $A_2$ is an interval-valued fuzzy set defined by

$$\begin{aligned} A_1\cap A_2= \left\{ \left( x, \left[ \min \{\mu _{A_1}^-(x), \mu _{A_2}^-(x)\},\min \{\mu _{A_1}^+(x),\mu _{A_2}^+(x)\}\right] \right) {:}\,x\in X\right\} . \end{aligned}$$

The fuzzy union of two IVFSs $A_1$ and $A_2$ is a IVFS defined by

$$\begin{aligned} A_1\cup A_2= \left\{ \left( x, \left[ \max \{\mu _{A_1}^-(x), \mu _{A_2}^-(x)\},\max \{\mu _{A_1}^+(x),\mu _{A_2}^+(x)\}\right] \right) {:}\,x\in X\right\} \end{aligned}$$

Fuzzy out-neighbourhood of a vertex $v\in V$ of an interval-valued fuzzy directed graph (IVFDG) $\overrightarrow{D}=(V,A,\overrightarrow{B})$ is the IVFS ${\mathcal {N}}^+(v)=(X_v^+, m_v^+)$, where $X_v^+=\{u{:}\, \mu _B(\overrightarrow{v,u})>0\}$ and $m_v^+{:}\,X_v^+\rightarrow [0,1]\times [0,1]$ defined by $m_v^+=\mu _B(\overrightarrow{v,u})=[\mu _B^-(\overrightarrow{v,u}), \mu _B^+(\overrightarrow{v,u})]$

Here, *B* is an interval-valued fuzzy relation on a set *X*, is denoted by $\mu _B{:}\,X\times X \rightarrow [0,1] \times [0,1]$ such that

$$\begin{aligned}&\mu _B^-(x,y)\le \min \left\{ \mu _A^-(x), \mu _A^-(y)\right\} \\&\mu _B^+(x,y)\le \min \left\{ \mu _A^+(x), \mu _A^+(y)\right\} \end{aligned}$$

An *interval-valued fuzzy graph* of a graph $G^*=(V,E)$ is a fuzzy graph $G=(V, A, B)$, where $A=[\mu _A^-, \mu _A^+]$ is an interval-valued fuzzy set on *V* and $B=[\mu _B^-, \mu _B^+]$ is a symmetric interval-valued fuzzy relation on *E*. An interval-valued fuzzy digraph $\overrightarrow{G}=(V, A, \overrightarrow{B})$ is an interval-valued fuzzy graph, where the fuzzy relation $\overrightarrow{B}$ is antisymmetric.

An interval-valued fuzzy graph $\xi = (A,B)$ is said to be *complete interval-valued fuzzy graph* if $\mu ^-(x,y)= \min \{\sigma ^-(x),\sigma ^-(y)\}$ and $\mu ^+(x,y)=$ $\min$ $\{\sigma ^+(x),$ $\sigma ^+(y)\}$, $\forall x,y\in V$. An interval-valued fuzzy graph is defined to be *bipartite*, if there exists two sets $V_1$ and $V_2$ such that the sets $V_1$ and $V_2$ are partitions of the vertex set *V*, where $\mu ^+(u,v)=0$ if $u,v\in V_1$ or $u, v \in V_2$ and $\mu ^+(v_1, v_2) > 0$ if $v_1\in V_1$ (or $V_2$) and $v_2 \in V_2$ (or $V_1$).

The *Cartesian product* (Akram and Dudek 2011) $G_1\times G_2$ of two interval-valued fuzzy graphs $G_1 =(V_1, A_1,B_1)$ and $G_2 = (V_2,A_2,B_2)$ is defined as a pair $(V_1\times V_2, A_1\times A_2,B_1\times B_2)$ such that

1. $$\left\{ \begin{array}{l} \mu _{A_1\times A_2}^-(x_1, x_2) = \min \{\mu _{A_1}^-(x_1), \mu _{A_2}^-(x_2)\}\\ \mu ^+_{A_1\times A_2}(x_1, x_2) = \min \{\mu ^+_{A_1}(x_1), \mu ^+_{A_2}(x_2)\} \end{array}\right\}$$ for all $$x_1\in V_1, x_2\in V_2$$,
2. $$\left\{ \begin{array}{l} \mu _{B_1\times B_2}^-((x,x_2),(x,y_2)) = \min \{\mu _{A_1}^-(x), \mu _{B_2}^-(x_2,y_2)\}\\ \mu _{B_1\times B_2}^+((x,x_2),(x,y_2)) = \min \{\mu _{A_1}^+(x), \mu _{B_2}^+(x_2,y_2)\} \end{array}\right\}$$ for all $x\in V_1$ and $(x_2, y_2)\in E_2$,
3. $$\left\{ \begin{array}{l} \mu _{B_1\times B_2}^-((x_1,y),(y_1,y)) = \min \{\mu _{B_1}^-(x_1,y_1), \mu _{A_2}^-(y)\}\\ \mu _{B_1\times B_2}^+((x_1,y),(y_1,y)) = \min \{\mu _{B_1}^+(x_1,y_1), \mu _{A_2}^+(y)\} \end{array}\right\}$$ for all $(x_1,y_1)\in E_1$ and $y \in V_2.$

The composition $G_1[G_2]=(V_1\circ V_2, A_1\circ A_2, B_1\circ B_2)$ of two interval-valued fuzzy graphs $G_1$ and $G_2$ of the graphs $G_1^*$ and $G_2^*$ is defined as follows:

1. $$\left\{ \begin{array}{l} \mu _{A_1\circ A_2}^-(x_1, x_2) = \min \{\mu _{A_1}^-(x_1), \mu _{A_2}^-(x_2)\}\\ \mu ^+_{A_1\circ A_2}(x_1, x_2) = \min \{\mu ^+_{A_1}(x_1), \mu ^+_{A_2}(x_2)\} \end{array}\right\}$$ for all $$x_1\in V_1, x_2\in V_2$$,
2. $$\left\{ \begin{array}{l} \mu _{B_1\circ B_2}^-((x,x_2),(x,y_2)) = \min \{\mu _{A_1}^-(x), \mu _{B_2}^-(x_2,y_2)\}\\ \mu _{B_1\circ B_2}^+((x,x_2),(x,y_2)) = \min \{\mu _{A_1}^+(x), \mu _{B_2}^+(x_2,y_2)\} \end{array}\right\}$$ for all $x\in V_1$ and $(x_2, y_2)\in E_2$,
3. $$\left\{ \begin{array}{l} \mu _{B_1\circ B_2}^-((x_1,y),(y_1,y)) = \min \{\mu _{B_1}^-(x_1,y_1), \mu _{A_2}^-(y)\}\\ \mu _{B_1\circ B_2}^+((x_1,y),(y_1,y)) = \min \{\mu _{B_1}^+(x_1,y_1), \mu _{A_2}^+(y)\} \end{array}\right\}$$ for all $(x_1,y_1)\in E_1$ and $y \in V_2,$
4. $$\left\{ \begin{array}{l} \mu _{B_1\circ B_2}^-((x_1,x_2),(y_1,y_2)) = \min \{\mu _{A_2}^-(x_2), \mu _{A_2}^-(y_2),\mu _{B_1}^-(x_1,y_1)\}\\ \mu _{B_1\circ B_2}^+((x_1,x_2),(y_1,y_2)) = \min \{\mu _{A_2}^+(x_2), \mu _{A_2}^+(y_2),\mu _{B_1}(x_1,y_1)\} \end{array}\right\}$$ otherwise.

The *union* $G_1\cup G_2=(V_1\cup V_2, A_1\cup A_2, B_1\cup B_2)$ of two interval-valued fuzzy graphs $G_1$ and $G_2$ of the graphs $G_1^*$ and $G_2^*$ is defined as follows:

1. $$\left\{ \begin{array}{l} \mu _{A_1\cup A_2}^-(x) =\mu _{A_1}^-(x) {\text { if }}\,x\in V_1 {\text { and }}\, x\notin V_2\\ \mu _{A_1\cup A_2}^-(x) =\mu _{A_2}^-(x) {\text { if }}\,x\in V_2 {\text { and }}\,x\notin V_1\\ \mu _{A_1\cup A_2}^-(x) =\max \{\mu _{A_1}^-(x), \mu _{A_2}^-(x)\}\,{\text { if }}\,x\in V_1\cap V_2. \end{array}\right.$$
2. $$\left\{ \begin{array}{l} \mu _{A_1\cup A_2}^+(x) =\mu _{A_1}^+(x) {\text { if }}\, x\in V_1 {\text { and }}\,x\notin V_2\\ \mu _{A_1\cup A_2}^+(x) =\mu _{A_2}^+(x) {\text { if }}\,x\in V_2 {\text { and }}\,x\notin V_1\\ \mu _{A_1\cup A_2}^+(x) =\max \{\mu _{A_1}^+(x), \mu _{A_2}^+(x)\} {\text { if }}\,x\in V_1\cap V_2. \end{array}\right.$$
3. $$\left\{ \begin{array}{l} \mu _{B_1\times B_2}^-(x,y) = \mu _{B_1}^-(x,y) {\text { if }}\,(x,y)\in E_1 {\text{and}}\,(x,y)\notin E_2\\ \mu _{B_1\times B_2}^-(x,y) = \mu _{B_2}^-(x,y) {\text{if}}\,(x,y)\in E_2 {\text{and}}\,(x,y)\notin E_1\\ \mu _{B_1\times B_2}^-(x,y) = \max \{\mu _{B_1}^-(x,y), \mu _{B_2}^-(x,y)\} {\text{if}}\,(x,y)\in E_1\cap E_2. \end{array}\right.$$
4. $$\left\{ \begin{array}{l} \mu _{B_1\times B_2}^+(x,y) = \mu _{B_1}^+(x,y) {\text{if}}\,(x,y)\in E_1 {\text{and}}\,(x,y)\notin E_2\\ \mu _{B_1\times B_2}^+(x,y) = \mu _{B_2}^+(x,y) {\text{if}}\,(x,y)\in E_2 {\text{and}}\,(x,y)\notin E_1\\ \mu _{B_1\times B_2}^+(x,y) = \max \{\mu _{B_1}^+(x,y), \mu _{B_2}^+(x,y)\} {\text{if}}\,(x,y)\in E_1\cap E_2. \end{array}\right.$$

The *join* $G_1+G_2=(V_1+V_2, A_1+A_2, B_1+B_2)$ of two interval-valued fuzzy graphs $G_1$ and $G_2$ of the graphs $G_1^*$ and $G_2^*$ is defined as follows:

1. $$\left\{ \begin{array}{l} \mu _{A_1+ A_2}^-(x) = (\mu _{A_1}^-\cup \mu _{A_2}^-)(x)\\ \mu _{A_1+ A_2}^+(x) = (\mu _{A_1}^+\cup \mu _{A_2}^+)(x) \end{array}\right\}$$ if $$x\in V_1\cup V_2$$,
2. $$\left\{ \begin{array}{l} \mu _{B_1+ B_2}^-(x,y) = (\mu _{B_1}^-\cup \mu _{B_2}^-)(x,y)\\ \mu _{B_1+ B_2}^+(x,y) = (\mu _{B_1}^+\cup \mu _{B_2}^+)(x,y) \end{array}\right\}$$ if $$(x,y)\in E_1\cap E_2$$,
3. $$\left\{ \begin{array}{l} \mu _{B_1+ B_2}^-(x,y) = \min \{\mu _{A_1}^-(x), \mu _{A_2}^-(y)\}\\ \mu _{B_1+ B_2}^+(x,y) = \min \{\mu _{A_1}^+(x), \mu _{A_2}^+(y)\} \end{array}\right\}$$ for all $(x,y)\in E'$, where $E'$ is the set of edges connecting the vertices of $V_1$ and $V_2$.

## Interval-valued fuzzy $\phi$-tolerance competition graph

In this section, the definition of interval-valued fuzzy $\phi$-tolerance competition graph is given and studied several properties.

### **Definition 1**

(*Interval-valued fuzzy* $\phi$-*tolerance competition graph* (*IVFPTCG*)) Let $\phi {:}\,N\times N\rightarrow N$ be a mapping, where *N* is a set of natural numbers. Interval-valued fuzzy $\phi$-tolerance competition graph of an interval-valued fuzzy directed graph (IVFDG) $\overrightarrow{D}=(V,A,\overrightarrow{B})$ is an undirected graph $ITC_{\phi }(\overrightarrow{D}) = (V,A, B')$ such that

$$\begin{aligned} \mu _{B'} (u,v) &= {} [\mu _{B'}^-(u,v), \mu _{B'}^+(u,v)]\\& = {} \left\{ \begin{array}{l} h({{\mathcal {N}}}^+(u)\cap {\mathcal {N}}^+(v)),\\ \,\quad \qquad \text{ if } c({\mathcal {N}}^+(u)\cap {\mathcal {N}}^+(v))\ge \phi \{c({\mathcal {T}}_u), c({\mathcal {T}}_v)\}\\ \frac{s({\mathcal {N}}^+(u)\cap {\mathcal {N}}^+(v))-\phi \{s({\mathcal {T}}_u), s({\mathcal {T}}_v))\}+1}{s({\mathcal {N}}^+(u)\cap {\mathcal {N}}^+(v))}\cdot h({\mathcal {N}}^+(u)\cap {\mathcal {N}}^+(v)),\\ \,\quad \qquad \text{ if } s({\mathcal {N}}^+(u)\cap {\mathcal {N}}^+(v))\ge \phi \{s({\mathcal {T}}_u), s({\mathcal {T}}_v)\}\\ 0, \,\quad \text{ otherwise. } \end{array} \right. \end{aligned}$$

where, ${\mathcal {T}}_u, {\mathcal {T}}_v$ are the fuzzy tolerances corresponding to *u* and *v*, respectively.

Taking $\phi$ as $\min$. An example of this graph is given below.

### *Example 1*

**Fig. 2:**
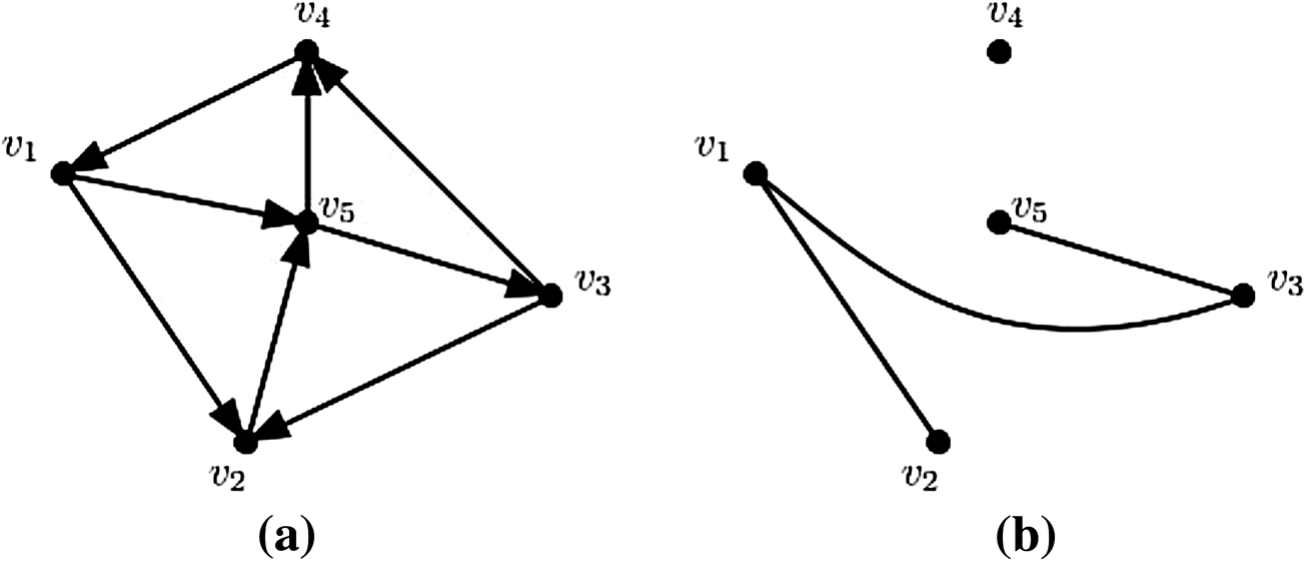
An interval-valued fuzzy digraph and its corresponding interval-valued fuzzy $\phi$-tolerance competition graph. **a** An interval-valued fuzzy digraph, **b** interval-valued fuzzy *ϕ*-tolerance competition graph

Consider an interval-valued fuzzy digraph $\overrightarrow{G}=(V,A,\overrightarrow{B})$ shown in Fig. 2 with each vertex have membership values [1, 1]. The edge membership values are taken as

$$\begin{aligned} &\mu _B(\overrightarrow{v_1,v_2})=[0.8,0.9], \quad \mu _B(\overrightarrow{v_1,v_5})=[0.7,0.8],\\ &\mu _B(\overrightarrow{v_2,v_5})=[0.6,0.8], \quad \mu _B(\overrightarrow{v_3,v_2})=[0.5,0.7],\\ &\mu _B(\overrightarrow{v_3,v_4})=[0.3,0.5], \quad \mu _B(\overrightarrow{v_4,v_1})=[0.7,0.9],\\ &\mu _B(\overrightarrow{v_5,v_3})=[0.6,0.8],\quad \mu _B(\overrightarrow{v_5,v_4})=[0.5,0.6]. \end{aligned}$$

Let core and support lengths of fuzzy tolerances ${\mathcal {T}}_1,{\mathcal {T}}_2, {\mathcal {T}}_3,{\mathcal {T}}_4,{\mathcal {T}}_5$ corresponding to the vertices $v_1, v_2,v_3,v_4,v_5$ be 1, 1, 3, 2, 0 and 1, 2, 4, 3, 1, respectively. Here, it is true that $\phi \{c({\mathcal {T}}_u), c({\mathcal {T}}_v)\}=\min \{c({\mathcal {T}}_u), c({\mathcal {T}}_v)\}$.

Based on this consideration, the following computations have been made.

$$\begin{aligned} {\mathcal {N}}^+(v_1)& = {} \{(v_2,[0.8,0.9]),(v_5,[0.7,0.8])\}\\ {\mathcal {N}}^+(v_2)& = {} \{(v_5,[0.6,0.8])\}\\ {\mathcal {N}}^+(v_3)& = {} \{(v_2,[0.5,0.7]),(v_4,[0.3,0.5])\}\\ {\mathcal {N}}^+(v_4)& = {} \{(v_1,[0.7,0.9])\}\\ {\mathcal {N}}^+(v_5)& = {} \{(v_3,[0.6,0.8]),(v_4,[0.5,0.6])\} \end{aligned}$$

Therefore,

$$\begin{aligned}&{\mathcal {N}}^+(v_1)\cap {\mathcal {N}}^+(v_2)=\{(v_5,[0.6,0.8])\}\\&{\mathcal {N}}^+(v_1)\cap {\mathcal {N}}^+(v_3)=\{(v_2,[0.5,0.7])\}\\&{\mathcal {N}}^+(v_3)\cap {\mathcal {N}}^+(v_5)=\{(v_4,[0.3,0.5])\} \end{aligned}$$

Then

$$\begin{aligned}&h({\mathcal {N}}^+(v_1)\cap {\mathcal {N}}^+(v_2))=[0.6,0.8]\\&h({\mathcal {N}}^+(v_1)\cap {\mathcal {N}}^+(v_3))=[0.5,0.7]\\&h({\mathcal {N}}^+(v_3)\cap {\mathcal {N}}^+(v_5))=[0.3,0.5] \end{aligned}$$

Now,

$$\begin{aligned}&c({\mathcal {N}}^+(v_1)\cap {\mathcal {N}}^+(v_2))=0; s({\mathcal {N}}^+(v_1)\cap {\mathcal {N}}^+(v_2))=1\\&c({\mathcal {N}}^+(v_1)\cap {\mathcal {N}}^+(v_3))=0; s({\mathcal {N}}^+(v_1)\cap {\mathcal {N}}^+(v_3))=1\\&c({\mathcal {N}}^+(v_3)\cap {\mathcal {N}}^+(v_5))=0; s({\mathcal {N}}^+(v_3)\cap {\mathcal {N}}^+(v_5))=1. \end{aligned}$$

Then by the definition of interval-valued fuzzy $\phi$-tolerance competition graph, the vertex membership function of the interval-valued fuzzy min-tolerance competition graph is that of interval-valued fuzzy digraph shown in Fig. 2 and the edge membership values are as follows:

$$\begin{aligned} \begin{array}{ll} \mu _B({v_1,v_3})=[0.5,0.7], &{}\quad \mu _B({v_1,v_2})=[0.6,0.8],\\ \mu _B({v_3,v_5})=[0.3,0.5]. \end{array} \end{aligned}$$

A $\phi$-T-edge clique cover ($\phi$-T-ECC) of an interval-valued fuzzy graph ${\mathcal {G}}=(V,A,B)$ with vertices $v_1,v_2,\ldots , v_n$ is a collection $S_1,S_2,\ldots , S_k$ of subsets of *V* such that $\mu _B^-(v_r,v_s)>0$ if and only if at least $\phi (c(T_r), c(T_s))$ of the sets $S_i$, contain both $v_r$ and $v_s$. The size *k* of a smallest $\phi$-T-ECC of ${\mathcal {G}}$ taken over all tolerances *T* is the $\phi$-T-edge clique cover number and is denoted by $\theta _{\phi }({\mathcal {G}})$.

### **Theorem 1**

*Let* $\phi {:}\,N\times N\rightarrow N$ *be a mapping. If* $\theta _{\phi }({\mathcal {G}})\le |V|$, *then there exists an interval-valued fuzzy* $\phi$ *-tolerance competition graph.*

### *Proof*

Let us assume that $\theta _{\phi }({\mathcal {G}})\le |V|$ and $S_1,S_2,\ldots , S_k (k\le n)$ be a $\phi$-T-ECC of an interval-valued fuzzy graph ${\mathcal {G}}$. Each $S_i$ is defined by $S_i=\{v_j{:}\,\mu _B^-(v_i, v_j)>0\}$. Each $S_i$ is chosen in such a way that in the interval-valued fuzzy digraph $\overrightarrow{{\mathcal {G}}}=(V,A,\overrightarrow{B})$, $\mu _B^-(\overrightarrow{v_i,v_j})=\mu _{B'}^-(v_i,v_j)$ and $\mu _B^+(\overrightarrow{v_i,v_j})=\mu _{B'}^+(v_i,v_j)$, if $v_j\in S_i$.

Now, in IVFG ${\mathcal {G}}$, either $c({\mathcal {N}}^+(v_i)\cap {\mathcal {N}}^+(v_j))\ge \phi \{c({\mathcal {T}}_{v_i}), c({\mathcal {T}}_{v_j})\}$ or, $s({\mathcal {N}}^+(v_i)\cap {\mathcal {N}}^+(v_j))\ge \phi \{s({\mathcal {T}}_{v_i}), s({\mathcal {T}}_{v_j})\}$ must satisfy.

Hence, ${\mathcal {G}}$ is an interval-valued fuzzy $\phi$-tolerance competition graph. $\square$

### **Theorem 2**

*For an interval-valued fuzzy digraph* ${\mathcal {G}}=(V,A,\overrightarrow{B})$, *if there exists an interval-valued fuzzy* $\phi$-*tolerance competition graph, then* $\theta _{\phi }(\overrightarrow{{\mathcal {G}}})\le |V|=n.$

### *Proof*

Let ${\mathcal {G}}=(V,A,B')$ be an interval-valued fuzzy $\phi$-tolerance competition graph of $\overrightarrow{G}$ and $V=\{v_1,v_2,\ldots , v_n\}$ and $S_i=\{v_j{:}\,\mu _{B'}^-(v_i,v_j)>0\}$. It is clear that there can be at most *n* numbers of $S_i$’s.

Let ${\mathcal {T}}_1,{\mathcal {T}}_2,\ldots , {\mathcal {T}}_n$ be the fuzzy tolerances associated to each vertex of *V*.

Now, $\mu (v_r,v_s)>0$ if and only if either $c({\mathcal {N}}^+(v_r)\cap {\mathcal {N}}^+(v_s))\ge \phi \{c({\mathcal {T}}_{r}), c({\mathcal {T}}_{s})\}$ or, $s({\mathcal {N}}^+(v_r)\cap {\mathcal {N}}^+(v_s))\ge \phi \{s({\mathcal {T}}_{r}), s({\mathcal {T}}_{s})\}$.

Thus, at most *n* sets $S_1,S_2,\ldots , S_n$ make a family of $\phi$-T-ECC of size at most $n=|V|$, i.e. $\theta _{\phi }(\overrightarrow{{\mathcal {G}}})\le |V|=n.$ $\square$

### **Theorem 3**

*Interval-valued fuzzy* $\phi$-*tolerance competition graph* $G=(V,A,B)$ *cannot be complete.*

### *Proof*

Suppose, *G* be an interval-valued fuzzy $\phi$-tolerance competition graph with 2 vertices, *x* and *y* (say). For this graph there is no interval digraph with 2 vertices with some common preys. Hence, it cannot be complete.

**Fig. 3:**
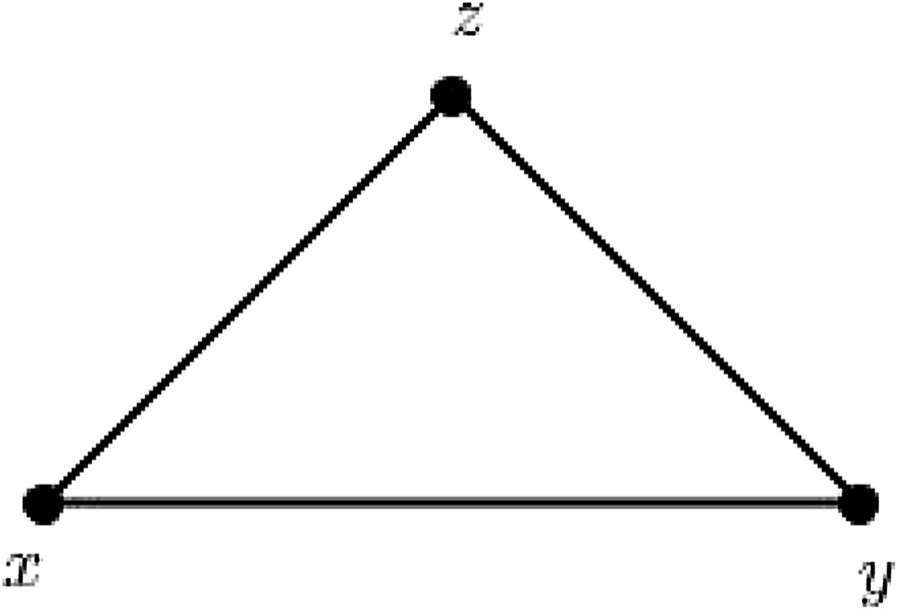
A complete IVFPTCG

If possible let, an IVFPTCG with 3 vertices be complete. Without any loss of generality, consider the graph of Fig. 3. This graph is nothing but a clique of order 3. As $\mu _B(x,y)\ne [0,0]$, *x*, *y* has a common prey and it must be *z*. Thus, *x*, *y* is directed to *z*. Again $\mu _B(y,z)\ne [0,0]$ implies that, *y*, *z* is directed to *x*. But in IVFDG, it is not possible to have two directed edges (*x*, *z*) and (*z*, *x*) simultaneously. This concludes that there is no valid IVFDG for this IVFPTCG.

As, every complete IVFPTCG contains a clique of order 3, there does not exist any valid IVFDG. Hence, any interval-valued fuzzy $\phi$-tolerance competition graph $G=(V,A,B)$ cannot be complete. $\square$

### *Remark 1*

The interval-valued fuzzy $\min$-tolerance competition graph of an irregular interval-valued fuzzy digraph need not be irregular.

**Fig. 4:**
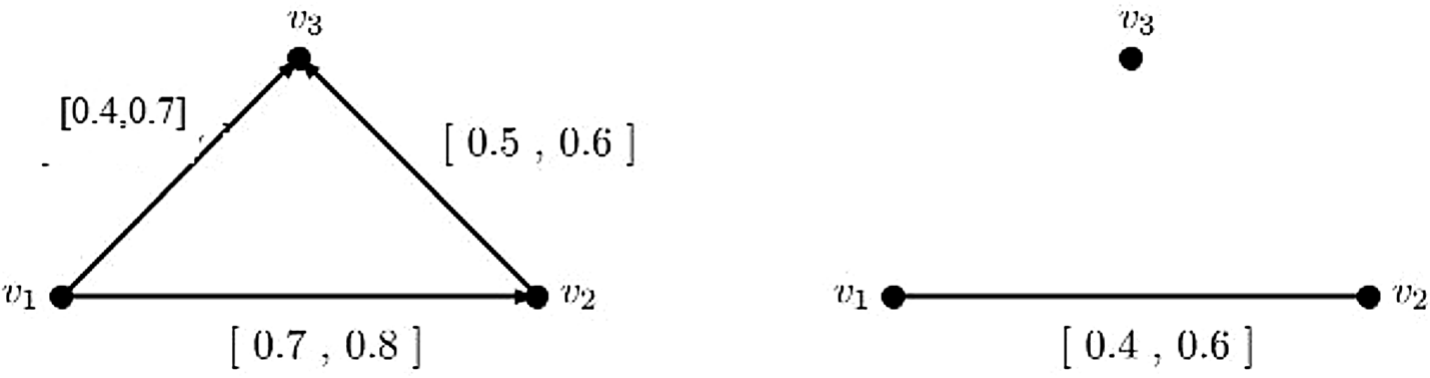
Irregular interval-valued fuzzy digraph and its corresponding interval-valued fuzzy min-tolerance competition graph

This can be shown by giving a counter-example. Suppose an interval-valued fuzzy digraph with 3 vertices shown in Fig. 4.

Consider the core and support lengths of fuzzy tolerances associated to each of the vertices of the irregular interval-valued fuzzy digraph shown in Fig. 4 are 1, 1, 1 and 1, 1, 1 respectively.

### *Remark 2*

The interval-valued fuzzy $\min$-tolerance competition graph of a regular interval-valued fuzzy digraph need not be regular.

**Fig. 5:**
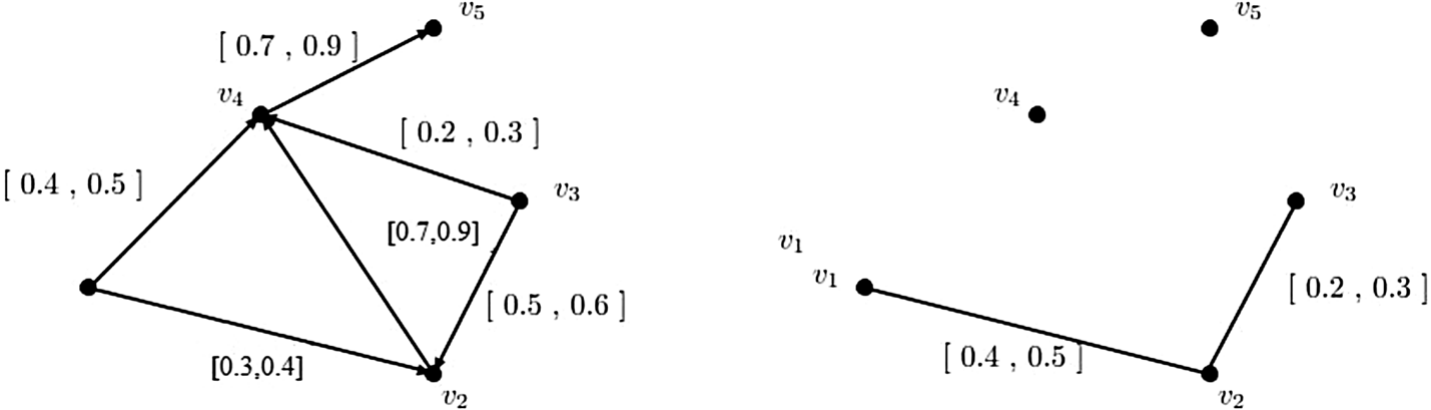
A regular interval-valued fuzzy digraph and its corresponding interval-valued fuzzy min-tolerance competition graph

To prove this, a counter-example is given in the Fig. 5.

In Fig. 5, the regular interval-valued fuzzy digraph has the degrees $\deg (v_1)=\deg (v_2)=\cdots = \deg (v_5)=[0.7,0.9]$, but the degree of the vertices of interval-valued fuzzy min-tolerance competition graph of the digraph shown in Fig. 5 are $\deg (v_1)=[0.4,0.5]$, $\deg (v_2)=[0.6,0.8]$, $\deg (v_3)=[0.2,0.3]$. Hence, it is not regular.

### **Definition 2**

The *size* of an interval-valued fuzzy graph ${\mathcal {G}}=(V,A, B)$ is denoted by $S({\mathcal {G}})$ and is defined by

$$\begin{aligned} S({\mathcal {G}})= \sum \mu _B(u,v)=\left[ \sum \mu _B^-(u,v), \sum \mu _B^+(u,v)\right] . \end{aligned}$$

### **Theorem 4**

*Let* $\overrightarrow{{\mathcal {G}}}$ *be an interval-valued fuzzy digraph and* $ITC_{\phi }(\overrightarrow{{\mathcal {G}}})$ *be its interval-valued fuzzy* $\phi$ *-tolerance competition graph. Then*

$$\begin{aligned} S(ITC_{\phi }(\overrightarrow{{\mathcal {G}}}))\le S(\overrightarrow{{\mathcal {G}}}). \end{aligned}$$

### *Proof*

Let $ITC_{\phi }(\overrightarrow{{\mathcal {G}}})=(V,A,B')$ be the interval-valued fuzzy $\phi$-tolerance competition graph of an interval-valued fuzzy digraph $\overrightarrow{{\mathcal {G}}}=(V,A,\overrightarrow{B})$. As for every triangular orientation of three vertices in $\overrightarrow{{\mathcal {G}}}$, as shown in Fig. 4, there is atmost one edge in $ITC_{\phi }(\overrightarrow{{\mathcal {G}}})$, it is obvious that, an interval-valued fuzzy $\phi$-tolerance competition graph has less number of edges than that of the interval-valued fuzzy digraph. Now, consider $\mu _{B'}(v_1,v_2)>0$ in $ITC_{\phi }(\overrightarrow{{\mathcal {G}}})$ and ${\mathcal {N}}^+(v_1)$ and ${\mathcal {N}}^+(v_2)$ has at least one vertex in common and also $h({\mathcal {N}}^+(v_1)\cap {\mathcal {N}}^+(v_2))=[1,1]$ (as much as possible). Then there exist at least one vertex, say $v_i$ so that the edge membership value between $v_1$, $v_i$ or $v_2$, $v_i$ is [1, 1]. Then $S(\overrightarrow{{\mathcal {G}}})>[1,1]$ whereas, $S(ITC_{\phi }(\overrightarrow{{\mathcal {G}}}))\le [1,1]$. Hence, $S(ITC_{\phi }(\overrightarrow{{\mathcal {G}}}))\le S(\overrightarrow{{\mathcal {G}}}).$ $\square$

### **Theorem 5**

*If* $C_1,C_2,\ldots , C_p$ *be the cliques of order* 3 *of underlying undirected crisp graph of a IVFDG* $\overrightarrow{G}=(V,A,\overrightarrow{B})$ *such that* $C_1\cup C_2\cup \ldots C_p=V$ *and* $|C_i\cap C_j|\le 1$ $\forall i,j=1,2,\ldots , p$. *Then the corresponding IVFPTCG of* $\overrightarrow{G}$ *cannot have cliques of order* 3 *or more.*

### *Proof*

**Fig. 6:**
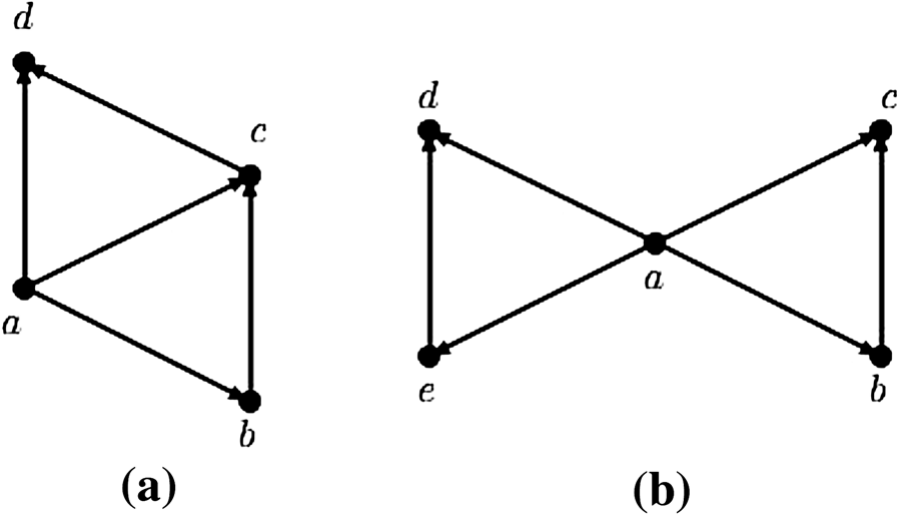
Types of triangular orientation. **a** Two triangular orientation has a common edge, **b** two triangular orientation has no common edge

From the given conditions of clique sets, i.e. $C_1\cup C_2\cup \ldots C_p=V$ and $|C_i\cap C_j|\le 1 \forall i,j=1,2,\ldots , p$, it is clear that the interval-valued fuzzy digraph has only triangular orientation and no two triangular orientation has a common edge. That is, the IVFDG has no orientation shown in Fig. 6b. The IVFDG only have the orientations of type shown in Fig. 6a.

As for every triangular orientation, there have only one edge in interval-valued fuzzy $\phi$-tolerance competition graph, the said graph does not have a clique of order 3 or more.

Hence, interval-valued fuzzy $\phi$-tolerance competition graph cannot have cliques of order 3 or more. $\square$

### **Theorem 6**

*If the clique number of an underlying undirected crisp graph of an interval-valued fuzzy digraph* $\overrightarrow{{\mathcal {G}}}=(V,A,\overrightarrow{B})$ *is* *p*, *then the underlying crisp graph of the interval-valued fuzzy* $\phi$-*tolerance competition graph has the clique number less than or equal to* *p*.

### *Proof*

Let us assume that the maximum clique of $\overrightarrow{{\mathcal {G}}}=(V,A,\overrightarrow{B})$ induces a subgraph $\overrightarrow{\mathcal {G'}}$ which is also an interval-valued fuzzy directed graph. From Theorem 4, the size of interval-valued fuzzy $\phi$-tolerance competition graph is always less than or equal to the size of interval-valued fuzzy directed graph, then the clique number of the interval-valued fuzzy $\phi$-tolerance competition graph cannot be greater than *p*. Hence the theorem follows.

### **Theorem 7**

*Interval-valued fuzzy* $\phi$-*tolerance competition graph of a complete interval-valued fuzzy digraph has maximum* $^nC_3$ *number of fuzzy edges.*

### *Proof*

It is obvious that every triangular orientation there exists an edge in IVFPTCG. Now, in a complete interval-valued fuzzy digraph $\mu _B^-(x,y)=\min \{\mu _A^-(x),\,\mu _A^-(y)\}$, and $\mu _B^+(x,y)=\min \{\mu _A^+(x),\mu _A^+(y)\}$, $\forall x, y \in V$. Hence, every vertex is assigned to some vertex in *V*. Therefore, there are maximum $^nC_3$ number of orientations. Therefore, there exists maximum $^nC_3$ number of fuzzy edges in IVFPTCG. $\square$

## Application of interval-valued fuzzy max-tolerance competition graph in image matching

**Fig. 7:**
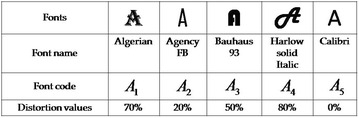
Different fonts of A and their distortion values

**Fig. 8:**
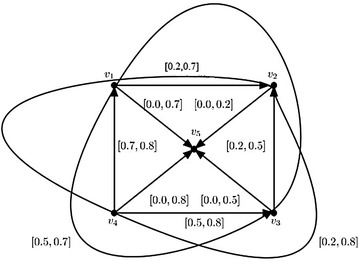
Interval-valued fuzzy digraph model of image matching

**Fig. 9:**
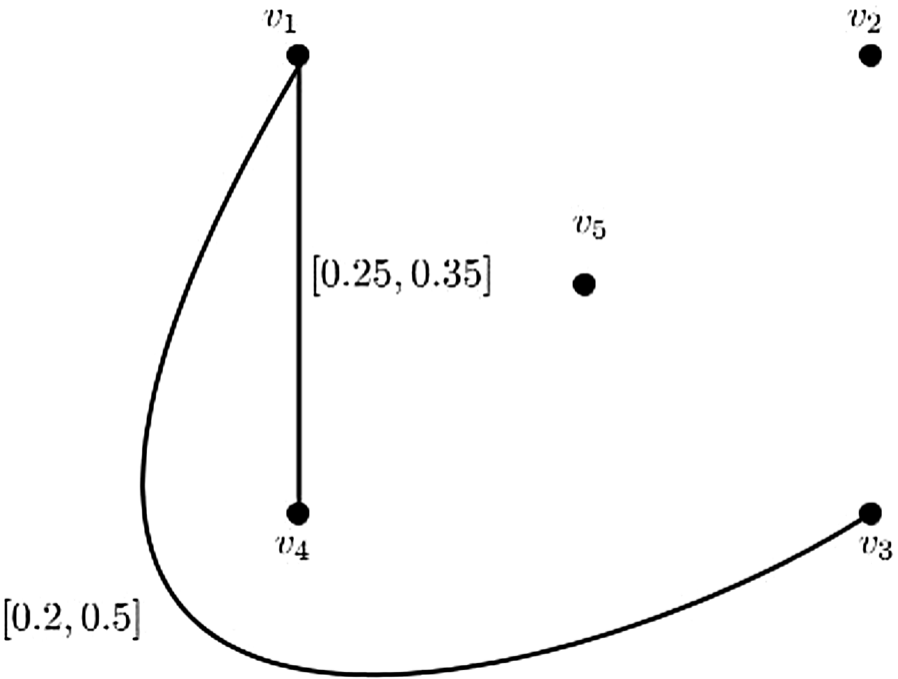
Interval-valued fuzzy max-tolerance competition graph of Fig. 8

Computer world advances rapidly in this modern age. Yet, it is till now a dull thing to us. The major difference for image matching by human and computer is that computer could not match two or more images by saying that they are likely same, but human can. Here, we present an arbitrary example by considering that the images are distorted by some way and they have some distortion values like an image of an object without 20% distorted (here, it is taken as arbitrary, it can be calculated by some pixel matching algorithm, which should be developed). For convenience, let us consider five types of different fonts $A_1,A_2,A_3,A_4,A_5$ of the alphabet *A* as shown in Fig. 7. Taking each fonts $A_1,A_2,A_3,A_4,A_5$ as vertices $v_1,v_2,v_3,v_4,v_5$ respectively and there exists an edge between the vertices if two fonts have two different distortion values (d.v.). The corresponding graph model is shown in Fig. 8. Let the distortion values of fonts $A_1,A_2,A_3,A_4,A_5$ be 70, 20, 50, 80, 0% respectively. This can be modeled as the interval-valued fuzzy digraph (see Fig. 8) with a direction to the vertex, which has the minimum distortion value. The edge membership value of an edge between two vertices $v_1$, $v_2$ of this graph is calculated as $\mu _B(v_1,v_2)=[\min \{\frac{\text {d.v. of }v_1}{100},$ $\frac{\text {d.v. of }v_2}{100}\},$ $\max \{\frac{\text {d.v. of }v_1}{100},\,\frac{\text {d.v. of }v_2}{100}\} ]$. Each fonts have some tolerances i. e., the fonts can be distorted to a certain percentage. Arbitrarily, let us consider the tolerance core and tolerance support lengths of the vertices $v_1,v_2,v_3,v_4,v_5$ are 0, 1, 0, 1, 2 and 1, 1, 1, 2, 3, respectively. Natural computations can be made and the max-tolerance competition graph is obtained as shown in Fig. 9, which shows that the fonts $A_1,A_4$ are closely related and the closeness is approximately $(0.35-0.25)\cdot 100\%=10\%$.

## Product of two IVFPTCGs and relations between them

Throughout this paper, $\theta$ is taken as the null set in crisp sense and $\overrightarrow{G_1^*}$, $\overrightarrow{G_2^*}$ are the crisp digraphs.

### **Definition 3**

The *Cartesian product* $G_1\times G_2$ of two interval-valued fuzzy digraphs $\overrightarrow{G_1} =(A_1,\overrightarrow{B_1})$ and $\overrightarrow{G_2} = (A_2,\overrightarrow{B_2})$ of the graphs $\overrightarrow{G^*_1} = (V_1,\overrightarrow{E_1})$ and $\overrightarrow{G^*_2} = (V_2,\overrightarrow{E_2})$ is defined as a pair $(A_1\times A_2,\overrightarrow{B_1\times B_2})$ such that

1. $$\left\{ \begin{array}{l} \mu _{A_1\times A_2}^-(x_1, x_2) = \min \{\mu _{A_1}^-(x_1), \mu _{A_2}^-(x_2)\}\\ \mu ^+_{A_1\times A_2}(x_1, x_2) = \min \{\mu ^+_{A_1}(x_1), \mu ^+_{A_2}(x_2)\} \end{array}\right\}$$ for all $$x_1\in V_1, x_2\in V_2$$,
2. $$\left\{ \begin{array}{l} \mu _{B_1\times B_2}^-(\overrightarrow{(x,x_2),(x,y_2)}) = \min \{\mu _{A_1}^-(x), \mu _{B_2}^-(\overrightarrow{x_2,y_2})\}\\ \mu _{B_1\times B_2}^+(\overrightarrow{(x,x_2),(x,y_2)}) = \min \{\mu _{A_1}^+(x), \mu _{B_2}^+(\overrightarrow{x_2,y_2})\} \end{array}\right\}$$ for all $x\in V_1$ and $(\overrightarrow{x_2, y_2})\in E_2$,
3. $$\left\{ \begin{array}{l} \mu _{B_1\times B_2}^-(\overrightarrow{(x_1,y),(y_1,y)}) = \min \{\mu _{B_1}^-(\overrightarrow{x_1,y_1}), \mu _{A_2}^-(y)\}\\ \mu _{B_1\times B_2}^+(\overrightarrow{(x_1,y),(y_1,y)}) = \min \{\mu _{B_1}^+(\overrightarrow{x_1,y_1}), \mu _{A_2}^+(y)\} \end{array}\right\}$$ for all $(\overrightarrow{x_1,y_1})\in E_1$ and $y \in V_2$.

### **Theorem 8**

*For any two interval-valued fuzzy directed graphs* $\overrightarrow{G_1}$ *and* $\overrightarrow{G_2}$,

$$\begin{aligned} ITC_{\phi }(\overrightarrow{G_1}\times \overrightarrow{G_2})= ITC_{\phi }(\overrightarrow{G_1})\times ITC_{\phi }(\overrightarrow{G_2}), \end{aligned}$$

*considering tolerances* ${\mathcal {T}}_{(x,y)}$ *corresponding to each vertex* (*x*, *y*) *of* $\overrightarrow{G_1}\times \overrightarrow{G_2}$ *as* $c({\mathcal {T}}_{(x,y)})=\min \{c({\mathcal {T}}_x),c({\mathcal {T}}_y)\}$ *and* $s({\mathcal {T}}_{(x,y)})=\min \{s({\mathcal {T}}_x),s({\mathcal {T}}_y)\}$.

### *Proof*

It is easy to understand from the definition of IVFPTCG that all vertices and their membership values remain unchanged, but fuzzy edges and their membership values have been changed. Thus, there is no need to clarify about vertices.

Now, according to the definition of *Cartesian product* of two interval-valued fuzzy directed graphs $\overrightarrow{G_1}$ and $\overrightarrow{G_2}$, there are two types of edges in $\overrightarrow{G_1}\times \overrightarrow{G_2}$. The two cases are as follows.

Suppose, all edges are of type $((x,x_2),(x,y_2))$, $\forall x\in V_1$ and $(x_2,y_2)\in E_2$.

Obviously, from the definition of the *Cartesian products* of two directed graphs that, if $x_2, y_2$ have a common prey $z_2$ in $\overrightarrow{G_2}$, then $(x,x_2),(x,y_2)$ have a common prey $(x,z_2)$ in $\overrightarrow{G_1}\times \overrightarrow{G_2}$, $\forall x\in V_1$. Now, it has to show if $\mu _{B_2}^-(x_2,y_2)>0$ in $ITC_{\phi }(\overrightarrow{G_2})$, then $\mu _{B_1\times B_2}^-((x,x_2),(x,y_2))>0$ in $ITC_{\phi }(\overrightarrow{G_1}$ $\times \overrightarrow{G_2})$ is true. If $\mu _{B_2}^-(x_2,y_2)$ $>0$, then either $c({\mathcal {N}}^+(x_2)\cap$ ${\mathcal {N}}^+(y_2))\ge$ $\phi \{c({\mathcal {T}}_{x_2}),$ $c({\mathcal {T}}_{y_2})\}$ or $s({\mathcal {N}}^+(x_2)\cap$ ${\mathcal {N}}^+(y_2))$ $\ge$ $\phi \{s({\mathcal {T}}_{x_2}),$ $s({\mathcal {T}}_{y_2})\}$ is true. From the previous claim, if $z_2$ is the common prey of $x_2, y_2$ in $\overrightarrow{G_2}$, $(x,z_2)$ is also a common prey of $(x,x_2)$ and $(x,y_2)$ in $\overrightarrow{G_1}\times \overrightarrow{G_2}$. Thus,

$$\begin{aligned} s({\mathcal {N}}^+(x,x_2)\cap {\mathcal {N}}^+(x,y_2))& = {} s\left( {\mathcal {N}}^+(x_2)\cap {\mathcal {N}}^+(y_2)\right) \\ &\ge \phi \left( s({\mathcal {T}}_{x_2}), s({\mathcal {T}}_{y_2})\right) \\ &\ge \phi \left( \min \left\{ s({\mathcal {T}}_x),s( {\mathcal {T}}_{x_2})\right\} ,\min \left\{ s({\mathcal {T}}_x),s({\mathcal {T}}_{y_2})\right\} \right) \\& = {} \phi \left( s({\mathcal {T}}_{(x,x_2)}), s({\mathcal {T}}_{(x,y_2)})\right) . \end{aligned}$$

As, the either case is satisfied, therefore $\mu _{B_1\times B_2}^-((x,x_2),(x,y_2))>0$.

If all edges of type $((x_1,y),(y_1,y))$, $\forall y\in V_2$ and $(x_1,y_1)\in E_1$, then the proof is similar to above case.

Hence, $ITC_{\phi }(\overrightarrow{G_1}\times \overrightarrow{G_2})= ITC_{\phi }(\overrightarrow{G_1})\times ITC_{\phi }(\overrightarrow{G_2})$ is proved. $\square$

### **Definition 4**

The composition $\overrightarrow{G_1}[\overrightarrow{G_2}]=(A_1\circ A_2, \overrightarrow{B_1\circ B_2})$ of two interval-valued fuzzy digraphs $\overrightarrow{G_1}$ and $\overrightarrow{G_2}$ of the graphs $\overrightarrow{G_1^*}$ and $\overrightarrow{G_2^*}$ is given as follows:

1. $$\left\{ \begin{array}{l} \mu _{A_1\circ A_2}^-(x_1, x_2) = \min \{\mu _{A_1}^-(x_1), \mu _{A_2}^-(x_2)\}\\ \mu ^+_{A_1\circ A_2}(x_1, x_2) = \min \{\mu ^+_{A_1}(x_1), \mu ^+_{A_2}(x_2)\} \end{array}\right\}$$ for all $$x_1\in V_1, x_2\in V_2$$,
2. $$\left\{ \begin{array}{l} \mu _{B_1\circ B_2}^-(\overrightarrow{(x,x_2),(x,y_2)}) = \min \{\mu _{A_1}^-(x), \mu _{B_2}^-(\overrightarrow{x_2,y_2})\}\\ \mu _{B_1\circ B_2}^+(\overrightarrow{(x,x_2),(x,y_2)}) = \min \{\mu _{A_1}^+(x), \mu _{B_2}^+(\overrightarrow{x_2,y_2})\} \end{array}\right\}$$ for all $x\in V_1$ and $(\overrightarrow{x_2, y_2})\in E_2$,
3. $$\left\{ \begin{array}{l} \mu _{B_1\circ B_2}^-(\overrightarrow{(x_1,y),(y_1,y)}) = \min \{\mu _{B_1}^-(\overrightarrow{x_1,y_1}), \mu _{A_2}^-(y)\}\\ \mu _{B_1\circ B_2}^+(\overrightarrow{(x_1,y),(y_1,y)}) = \min \{\mu _{B_1}^+(\overrightarrow{x_1,y_1}), \mu _{A_2}^+(y)\} \end{array}\right\}$$ for all $(\overrightarrow{x_1,y_1})\in E_1$ and $y \in V_2$
4. $$\left\{ \begin{array}{l} \mu _{B_1\circ B_2}^-(\overrightarrow{(x_1,x_2),(y_1,y_2)}) = \min \{\mu _{A_2}^-(x_2), \mu _{A_2}^-(y_2),\mu _{B_1}^-(\overrightarrow{x_1,y_1})\}\\ \mu _{B_1\circ B_2}^+(\overrightarrow{(x_1,x_2),(y_1,y_2)}) = \min \{\mu _{A_2}^+(x_2), \mu _{A_2}^+(y_2),\mu _{B_1}(\overrightarrow{x_1,y_1})\} \end{array}\right\}$$ otherwise.

### **Theorem 9**

*For any two interval-valued fuzzy directed graphs* $\overrightarrow{G_1}$ *and* $\overrightarrow{G_2}$,

$$\begin{aligned} ITC_{\phi }(\overrightarrow{G_1}\circ \overrightarrow{G_2})= ITC_{\phi }(\overrightarrow{G_1})\circ ITC_{\phi }(\overrightarrow{G_2}), \end{aligned}$$

*considering tolerances* ${\mathcal {T}}_{(x,y)}$ *corresponding to each vertices* (*x*, *y*) *of* $\overrightarrow{G_1}\circ \overrightarrow{G_2}$ *as* $c({\mathcal {T}}_{(x,y)})=\min \{c({\mathcal {T}}_x),c({\mathcal {T}}_y)\}$ *and* $s({\mathcal {T}}_{(x,y)})=\min \{s({\mathcal {T}}_x),s({\mathcal {T}}_y)\}$.

### *Proof*

According to the same interpretation drawn in Theorem 8, the membership values of the vertices of $\overrightarrow{G_1}[\overrightarrow{G_2}]$ remains unchanged under the composition $\circ$.

Now, according to the definition of composition $\overrightarrow{G_1}[\overrightarrow{G_2}]=(A_1\circ A_2, B_1\circ B_2)$ of two interval-valued fuzzy directed graphs $\overrightarrow{G_1}$ and $\overrightarrow{G_2}$, there are three types of edges in $\overrightarrow{G_1}\circ \overrightarrow{G_2}$. The three cases are as follows:

**Case I**: For all edges of type $((x,x_2),(x,y_2))$, $\forall x\in V_1$ and $(x_2,y_2)\in E_2$.
  Obviously, from the definition of the *Cartesian products* of two directed graphs that, if $x_2, y_2$ have a common prey $z_2$ in $\overrightarrow{G_2}$ then, $(x,x_2),(x,y_2)$ have also a common prey $(x,z_2)$ in $\overrightarrow{G_1}\circ \overrightarrow{G_2}$, $\forall x\in V_1$. Now, if $\mu _{B_2}^-(x_2,y_2)>0$ in $ITC_{\phi }(\overrightarrow{G_2})$, then $\mu _{B_1\circ B_2}^-((x,x_2),(x,y_2))>0$ in $ITC_{\phi }(\overrightarrow{G_1}\circ \overrightarrow{G_2})$. If $\mu _{B_2}^-(x_2,y_2)>0$, then either $c({\mathcal {N}}^+(x_2)\cap {\mathcal {N}}^+(y_2))\ge \phi \{c({\mathcal {T}}_{x_2}), c({\mathcal {T}}_{y_2})\}$ or $s({\mathcal {N}}^+(x_2)\cap {\mathcal {N}}^+(y_2))\ge \phi \{s({\mathcal {T}}_{x_2}), s({\mathcal {T}}_{y_2})\}$ is true. From the previous claim that if $z_2$ is the common prey of $x_2, y_2$ in $\overrightarrow{G_2}$, $(x,z_2)$ is also a common prey of $(x,x_2)$ and $(x,y_2)$ in $\overrightarrow{G_1}\circ \overrightarrow{G_2}$, then
  $$\begin{aligned} s({\mathcal {N}}^+(x,x_2)\cap {\mathcal {N}}^+(x,y_2))& = {} s({\mathcal {N}}^+(x_2)\cap {\mathcal {N}}^+(y_2))\\ &\ge \phi (s({\mathcal {T}}_{x_2}), s({\mathcal {T}}_{y_2}))\\ &\ge \phi (\min \{s({\mathcal {T}}_x),s( {\mathcal {T}}_{x_2})\},\min \{s({\mathcal {T}}_x),s({\mathcal {T}}_{y_2})\})\\& = {} \phi (s({\mathcal {T}}_{(x,x_2)}), s({\mathcal {T}}_{(x,y_2)})). \end{aligned}$$
  As, the either case is satisfied, $\mu _{B_1\circ B_2}((x,x_2),(x,y_2))>0$ is true.
**Case II**: For all edges of type $((x_1,y),(y_1,y))$, $\forall y\in V_2$ and $(x_1,y_1)\in E_1$.
  This is similar as the Case I.
**Case III**: For all edges of type $((x_1,x_2),(y_1,y_2))$, where $x_1\ne y_1$ and $x_2\ne y_2$.
  In this case, $(x_1,x_2)$ and $(y_1,y_2)$ have a common prey $(z_1,z_2)$ in $\overrightarrow{G_1}\circ \overrightarrow{G_2}$ if $x_1, y_1$ has a common prey $z_1$ in $\overrightarrow{G_1}$. In the similar way as in Case I, we can obtain
  $$\begin{aligned} s\left( {\mathcal {N}}^+(x_1,x_2)\cap {\mathcal {N}}^+(y_1,y_2)\right)& = {} s\left( {\mathcal {N}}^+(x_1)\cap {\mathcal {N}}^+(y_1)\right) \\ &\ge \phi \left( s({\mathcal {T}}_{x_1}), s({\mathcal {T}}_{y_1})\right) \\ &\ge \phi \left( \min \{s({\mathcal {T}}_{x_1}),s( {\mathcal {T}}_{x_2})\},\min \left\{ s({\mathcal {T}}_{y_1}),s({\mathcal {T}}_{y_2})\right\} \right) \\& = {} \phi \left( s({\mathcal {T}}_{(x_1,x_2)}), s({\mathcal {T}}_{(y_1,y_2)})\right) . \end{aligned}$$
  If, either case is satisfied, then $\mu _{B_1\circ B_2}^-((x_1,x_2),(y_1,y_2))>0$ is valid.

Hence, $ITC_{\phi }(\overrightarrow{G_1}\circ \overrightarrow{G_2})= ITC_{\phi }(\overrightarrow{G_1})\circ ITC_{\phi }(\overrightarrow{G_2})$ is proved. $\square$

### **Definition 5**

The *union* $\overrightarrow{G_1}\cup \overrightarrow{G_2}=(A_1\cup A_2, \overrightarrow{B_1\cup B_2})$ of two interval-valued fuzzy digraphs $\overrightarrow{G_1}$ and $\overrightarrow{G_2}$ of the graphs $\overrightarrow{G_1^*}$ and $\overrightarrow{G_2^*}$ is defined as follows:

1. $$\left\{ \begin{array}{l} \mu _{A_1\cup A_2}^-(x) =\mu _{A_1}^-(x) {\text{if}}\,x\in V_1 {\hbox{and}} x\notin V_2\\ \mu _{A_1\cup A_2}^-(x) =\mu _{A_2}^-(x) {\text{if}}\,x\in V_2 {\hbox{and}} x\notin V_1\\ \mu _{A_1\cup A_2}^-(x) =\max \{\mu _{A_1}^-(x), \mu _{A_2}^-(x)\} {\text{if}}\,x\in V_1\cap V_2. \end{array}\right.$$
2. $$\left\{ \begin{array}{l} \mu _{A_1\cup A_2}^+(x) =\mu _{A_1}^+(x) {\text{if}}\,x\in V_1 {\hbox{and}} x\notin V_2\\ \mu _{A_1\cup A_2}^+(x) =\mu _{A_2}^+(x) {\text{if}}\,x\in V_2 {\hbox{and}} x\notin V_1\\ \mu _{A_1\cup A_2}^+(x) =\max \{\mu _{A_1}^+(x), \mu _{A_2}^+(x)\} {\text{if}}\,x\in V_1\cap V_2. \end{array}\right.$$
3. $$\left\{ \begin{array}{l} \mu _{B_1\times B_2}^-(\overrightarrow{x,y}) = \mu _{B_1}^-(\overrightarrow{x,y}) {\text{if}}\,(\overrightarrow{x,y})\in E_1 {\text{and}}\,(\overrightarrow{x,y})\notin E_2\\ \mu _{B_1\times B_2}^-(\overrightarrow{x,y}) = \mu _{B_2}^-(\overrightarrow{x,y}) {\text{if}}\,(\overrightarrow{x,y})\in E_2 {\text{and}}\,(\overrightarrow{x,y})\notin E_1\\ \mu _{B_1\times B_2}^-(\overrightarrow{x,y}) = \max \{\mu _{B_1}^-(\overrightarrow{x,y}), \mu _{B_2}^-(\overrightarrow{x,y})\} {\text{if}}\,(\overrightarrow{x,y})\in E_1\cap E_2. \end{array}\right.$$
4. $$\left\{ \begin{array}{l} \mu _{B_1\times B_2}^+(\overrightarrow{x,y}) = \mu _{B_1}^+(\overrightarrow{x,y}) {\text{if}}\,(\overrightarrow{x,y})\in E_1 {\text{and}}\,(\overrightarrow{x,y})\notin E_2\\ \mu _{B_1\times B_2}^+(\overrightarrow{x,y}) = \mu _{B_2}^+(\overrightarrow{x,y}) {\text{if}}\,(\overrightarrow{x,y})\in E_2 {\text{and}}\,(\overrightarrow{x,y})\notin E_1\\ \mu _{B_1\times B_2}^+(\overrightarrow{x,y}) = \max \{\mu _{B_1}^+(\overrightarrow{x,y}), \mu _{B_2}^+(\overrightarrow{x,y})\} {\text{if}}\,(\overrightarrow{x,y})\in E_1\cap E_2. \end{array}\right.$$

### **Theorem 10**

*For any two interval-valued fuzzy directed graphs* $\overrightarrow{G_1}$ *and* $\overrightarrow{G_2}$,

$$\begin{aligned} ITC_{\phi }(\overrightarrow{G_1}\cup \overrightarrow{G_2})= ITC_{\phi }(\overrightarrow{G_1})\cup ITC_{\phi }(\overrightarrow{G_2}). \end{aligned}$$

### *Proof*

There are four cases as follows:

**Case I**: $$V_1\cap V_2=\theta$$
  In this case, $\overrightarrow{G_1}\cup \overrightarrow{G_2}$ is a disconnected interval-valued fuzzy directed graphs with two components $\overrightarrow{G_1}$ and $\overrightarrow{G_2}$. Thus, there is nothing to prove that $ITC_{\phi }(\overrightarrow{G_1}\cup \overrightarrow{G_2})= ITC_{\phi }(\overrightarrow{G_1})\cup ITC_{\phi }(\overrightarrow{G_2}).$
**Case II**: $V_1\cap V_2=\theta$, $(x_1,x_2)\in E_1$ and $(x_1,x_2)\notin E_2$
$\mu _{B_1\cup B_2}^-(x_1,x_2)=\mu _{B_1}^-(x_1,x_2)$ and it is obvious that if $\mu _{B_1}^-(x_1,x_2)>0$ in $ITC_{\phi }(\overrightarrow{G_1})$, then $\mu _{B_1\cup B_2}^-(x_1,x_2)>0$ in $ITC_{\phi }(\overrightarrow{G_1}\cup \overrightarrow{G_2})$.
**Case III**: $V_1\cap V_2=\theta$, $(x_1,x_2)\notin E_1$ and $(x_1,x_2)\in E_2$
  This is similar as in Case II.
**Case IV**: $$V_1\cap V_2=\theta$$, $$(x_1,x_2)\in E_1\cap E_2$$
  In this case, consider $x_1$ and $x_2$ have a common prey $y_1$ in $\overrightarrow{G_1}$ and $y_2$ in $\overrightarrow{G_2}$. This shows that $s({\mathcal {N}}^+(x_1)\cap {\mathcal {N}}^+(x_2))$ in $\overrightarrow{G_1}\cup \overrightarrow{G_2}$ is greater than or equal to $s({\mathcal {N}}^+(x_1)\cap {\mathcal {N}}^+(x_2))$ in $\overrightarrow{G_1}$ or $\overrightarrow{G_2}$. Hence, it can be found that if $\mu _{B_1}^-(x_1,x_2)>0$ in $ITC_{\phi }(\overrightarrow{G_1})$ and $\mu _{B_2}^-(x_1,x_2)>0$ in $ITC_{\phi }(\overrightarrow{G_2})$, then $\mu _{B_1\cup B_2}^-(x_1,x_2)>0$ in $ITC_{\phi }(\overrightarrow{G_1}\cup \overrightarrow{G_2})$.

Hence, $ITC_{\phi }(\overrightarrow{G_1}\cup \overrightarrow{G_2})= ITC_{\phi }(\overrightarrow{G_1})\cup ITC_{\phi }(\overrightarrow{G_2})$ is proved. $\square$

### **Definition 6**

The *join* $\overrightarrow{G_1}+\overrightarrow{G_2}=(A_1+A_2, \overrightarrow{B_1+B_2})$ of two interval-valued fuzzy digraphs $\overrightarrow{G_1}$ and $\overrightarrow{G_2}$ of the graphs $\overrightarrow{G_1^*}$ and $\overrightarrow{G_2^*}$ is defined as follows:

1. $$\left\{ \begin{array}{l} \mu _{A_1+ A_2}^-(x) = (\mu _{A_1}^-\cup \mu _{A_2}^-)(x)\\ \mu _{A_1+ A_2}^+(x) = (\mu _{A_1}^+\cup \mu _{A_2}^+)(x) \end{array}\right\}$$ if $$x\in V_1\cup V_2$$,
2. $$\left\{ \begin{array}{l} \mu _{B_1+ B_2}^-(\overrightarrow{x,y}) = (\mu _{B_1}^-\cup \mu _{B_2}^-)(\overrightarrow{x,y})\\ \mu _{B_1+ B_2}^+(\overrightarrow{x,y}) = (\mu _{B_1}^+\cup \mu _{B_2}^+)(\overrightarrow{x,y}) \end{array}\right\}$$ if $$(\overrightarrow{x,y})\in E_1\cap E_2$$,
3. $$\left\{ \begin{array}{l} \mu _{B_1+ B_2}^-(\overrightarrow{x,y}) = \min \{\mu _{A_1}^-(x), \mu _{A_2}^-(y)\}\\ \mu _{B_1+ B_2}^+(\overrightarrow{x,y}) = \min \{\mu _{A_1}^+(x), \mu _{A_2}^+(y)\} \end{array}\right\}$$ for all $(\overrightarrow{x,y})\in E'$, where $E'$ is the set of edges connecting the vertices (nodes) of $V_1$ and $V_2$.

### **Theorem 11**

*For any two interval-valued fuzzy directed graphs* $\overrightarrow{G_1}$ *and* $\overrightarrow{G_2}$, $ITC_{\phi }(\overrightarrow{G_1}+ \overrightarrow{G_2})$ *has less* *number of edges than that in* $ITC_{\phi }(\overrightarrow{G_1})+ ITC_{\phi }(\overrightarrow{G_2}).$

### Proof

In $ITC_{\phi }(\overrightarrow{G_1})+ITC_{\phi }(\overrightarrow{G_2})$, $(\mu _{B_1}^-+\mu _{B_2}^-)(x_1,x_2)>0$ is true for all $x_1\in V_1$ and $x_2\in V_2$. But, in $\overrightarrow{G_1}+\overrightarrow{G_2}$, $x_1$ and $x_2$ have no common prey, then $\mu _{B_1+B_2}^-(x_1,x_2)=0$ is valid for all $x_1\in V_1$ and $x_2\in V_2$. Thus, for all $x_1, x_2\in V_1 \cup V_2$, $\mu _{B_1+B_2}^-(x_1,x_2)=0<(\mu _{B_1}^-+\mu _{B_2}^-)(x_1,x_2)$ is true always. Hence, the result follows. $\square$

## Insights of this study

- Interval-valued fuzzy $\phi$-tolerance competition graphs are introduced. The real life competitions in food web are perfectly represented by interval-valued fuzzy $\phi$-tolerance competition graphs.
- An application of fuzzy $\phi$-tolerance competition graph on image matching is provided. Particularly, interval-valued fuzzy max-tolerance competition graph is used for this. Here, distorted images are matched for computer usages.
- Product of two IVFPTCGs and relations between them are defined. These results will develop the theory of interval-valued fuzzy graph literature. Some important results (Theorem 2, 3, 5, 9, 10) are proved.

## Conclusions

Adding more uncertainty to fuzzy $\phi$-tolerance competition graph, the interval-valued fuzzy $\phi$-tolerance competition graph was introduced here. Some interesting properties was investigated. Interesting properties of the IVFPTCG were proved such that the IVFPTCG of a IVFDG behaved like a homomorphic function under some operations. Generally, competition graphs represent some competitions in food webs. But, it can be also used in every competitive systems. These competitive systems can be represented by bipolar fuzzy graphs, intuitionistic fuzzy graphs, etc. But, interval valued fuzzy sets are perfect to represent uncertainties. An application of IVFPTCG in image matching was illustrated. Also, it can be applied in various types of fields such as database management system, network designing, neural network, image searching in computer application, etc.

## References

[CR1] Akram M, Dudek WA (2011). Interval valued fuzzy graphs. Comput Math Appl.

[CR2] Bhutani KR, Battou A (2003). On M-strong fuzzy graphs. Inf Sci.

[CR3] Bhutani KR, Rosenfeld A (2003). Strong arcs in fuzzy graphs. Inf Sci.

[CR4] Brigham RC, McMorris FR, Vitray RP (1995). Tolerance competition graphs. Linear Algebra Appl.

[CR5] Cho HH, Kim SR, Nam Y (2000). The $$m$$-step competition graph of a digraph. Discrete Appl Math.

[CR6] Cohen JE (1968). Interval graphs and food webs: a finding and a problem, Document 17696-PR.

[CR7] Golumbic MC, Monma CL (1982) A generalization of interval graphs with tolerances. In: Proceedings of the 13th Southeastern conference on combinatories, graph theory and computing, Congressus Numerantium Utilitas Math, Winnipeg, pp 321–331

[CR8] Kauffman A (1973). Introduction a la Theorie des Sous-emsembles Flous.

[CR9] Kim SR, McKee TA, McMorris FR, Roberts FS (1995). $$p$$-competition graphs. Discrete Appl Math.

[CR10] Koczy LT (1992). Fuzzy graphs in the evaluation and optimization of networks. Fuzzy Sets Syst.

[CR11] Mathew S, Sunitha MS (2009). Types of arcs in a fuzzy graph. Inf Sci.

[CR12] Mordeson JN, Nair PS (2000). Fuzzy graphs and fuzzy hypergraphs.

[CR13] Pramanik T, Samanta S, Pal M (2014). Interval-valued fuzzy planar graphs. Int J Mach Learn Cybern.

[CR14] Pramanik T, Samanta S, Sarkar B, Pal M (2016). Fuzzy phi-tolerance competition graphs. Soft Comput.

[CR15] Samanta S, Pal M (2015). Fuzzy planar graphs. IEEE Trans Fuzzy Syst.

[CR16] Samanta S, Pal M, Akram M (2014). $$m$$-step fuzzy competition graphs. J Appl Math Comput.

[CR17] Samanta S, Pal M (2013). Fuzzy $$k$$-competition graphs and $$p$$-competition fuzzy graphs. Fuzzy Eng Inf.

[CR18] Samanta S, Pal M (2011). Fuzzy tolerance graphs. Int J Latest Trends Math.

[CR19] Rosenfeld A, Zadeh LA, Fu KS, Shimura M (1975). Fuzzy graphs. Fuzzy sets and their applications.

